# Tailoring phononic, electronic, and thermoelectric properties of orthorhombic GeSe through hydrostatic pressure

**DOI:** 10.1038/s41598-019-45949-8

**Published:** 2019-07-01

**Authors:** Kunpeng Yuan, Zhehao Sun, Xiaoliang Zhang, Dawei Tang

**Affiliations:** Key Laboratory of Ocean Energy Utilization and Energy Conservation of Ministry of Education, School of Energy and Power Engineering, Dalian University of Technology, Dalian, 116024 China

**Keywords:** Thermoelectric devices and materials, Electronic properties and materials

## Abstract

In this paper, we systematically investigate the effect of hydrostatic pressure on the phononic and electronic transport properties of orthorhombic *p*-type GeSe using first-principles based Boltzmann transport equation approach. It is found that the lattice thermal conductivities along the *a* and *c* directions increase with pressure, whereas it experiences a decrease along the *b* direction. This anomalous pressure dependent lattice thermal conductivity is attributed to the combined effect of enhanced phonon group velocity and reduced phonon lifetime. Additionally, the optical phonon branches have remarkable contributions to the total lattice thermal conductivity. The electronic transport calculations indicate that the Seebeck coefficient undergoes a sign change from *p*-type to *n*-type along the *a* direction under pressure, and a dramatic enhancement of the power factor is observed due to the boost of electrical conductivity. The predicted *ZT* values along the *a*, *b*, and *c* directions are 1.54, 1.09, and 1.01 at 700 K and 8 GPa, respectively, which are about 14, 7.3, and 1.9 times higher than those at zero pressure at experimental carrier concentration of ~10^18^ cm^−3^. Our study is expected to provide a guide for further optimization of the thermal and charge transport properties through hydrostatic pressure.

## Introduction

Thermoelectric materials, enabling a direct and reversible conversion between thermal energy and electricity based on the Seebeck effect and Peltier effect, play a vital role in the development of sustainable energy technologies. The wide applications of thermoelectric materials can effectively promote the energy utilization efficiency. The conversion efficiency of thermoelectric materials is characterized by the dimensionless figure of merit *ZT* = *Sσ*^2^*T*/*κ*, which relies on the Seebeck coefficient *S*, electrical conductivity *σ*, and thermal conductivity *κ* consisting of electronic and lattice contributions. Obviously, a high *ZT* value can be achieved by increasing the power factor (PF) *Sσ*^2^ or decreasing the thermal conductivity. However, owing to the coupling among Seebeck coefficient, electrical conductivity, and electronic thermal conductivity, it is extremely challenging to improve the Seebeck coefficient and electrical conductivity while reducing the electronic thermal conductivity simultaneously^1^. Among these thermoelectric parameters, lattice thermal conductivity is the only independent material property, thus the strategy of reducing lattice thermal conductivity is widely used to develop advanced thermoelectric materials^2,3^.

In recent years, SnSe, adopting a simple layered orthorhombic crystal structure, has attracted considerable attention from the scientific community as a promising thermoelectric material. Zhao *et al*.^4^ initially reported an unprecedented *ZT* of 2.6 ± 0.3 at 923 K in undoped single crystal SnSe. Such outstanding thermoelectric performance arises from its intrinsically ultralow lattice thermal conductivity due to strong anharmonicity in bonding. As a member of IV–VI compound, GeSe possesses the same layered structure as SnSe^5^. Theoretical calculations have predicted that GeSe should be a potential candidate for high-efficient thermoelectric materials. Assuming the same hole concentration with SnSe in the range of 4 × 10^19^–6.5 × 10^19^ cm^−3^, Hao *et al*.^6^ predicted an extremely high thermoelectric performance in hole-doped GeSe along the crystallographic *b*-axis with a *ZT* ranging from 0.8 at 300 K to 2.5 at 800 K, even superior to the SnSe compound. However, the optimization of carrier concentration by chemical doping for GeSe has proved challenging^7,8^. In order to increase its carrier concentration by doping, various elements, such as Cu, Ag and Na for *p*-type as well as Bi, Sb, La, As and I for *n*-type, have been tested, and only Ag-substitution on Ge site enabled a hole concentration up to ~10^18^ cm^−3^. At a carrier concentration of ~10^18^ cm^−3^, the maximal available *ZT* is only about 0.2 at 700 K for Ag-doped polycrystalline Ge_0.79_Ag_0.01_Sn_0.2_Se^8^.

Applying hydrostatic pressure has been shown as an effective way to modulate the electronic and phononic transport properties. Pressure has a strong influence on the electronic band structures, especially in the vicinity of the energy band gap. Since the Seebeck coefficient and electrical conductivity are strongly dependent on the electronic band structures, pressure can be used as a powerful tool to optimize the thermoelectric transport properties through the modification of the electronic band structures. Previous works have reported a remarkable enhancement of thermoelectric properties under high pressure either by pressure-enhanced electrical conductivity or by improved Seebeck coefficient^9–19^. Specifically, for the SnSe crystal, pressure significantly enhances the thermoelectric transport properties along all three directions in moderate temperature ranges due to the electrical transport boost^15^, which gives us a hint that the thermoelectric properties of GeSe may be effectively tailored by external pressure. Moreover, uniaxial or biaxial strain offer an alternative realization of pressure and have been shown to enhance the thermoelectric performance effectively^20,21^. In addition to the change of the electronic band structures, the phonon dispersion curves can be modified under high pressure, leading to the manipulation of phonon behaviors. Recently, the negative correlation between lattice thermal conductivity and pressure has been reported^22–24^. The decrease of the lattice thermal conductivity with the increase of pressure provides a routine to decouple electronic and phononic transport, which enlightens the strategy of minimizing the lattice thermal conductivity by pressure without deterioration in electrical conductivity for better thermoelectric performance. As a promising thermoelectric material with potential *ZT* value exceeding that of SnSe, the influence of pressure on the thermoelectric properties of GeSe is still unknown. Meanwhile, most previous theoretical works on the pressure dependence of the thermoelectric properties calculated the lattice thermal conductivity based on the Debye-Callaway model^6,25^, which ignores the contributions of optical phonon modes, and the effect of pressure on the phonon transport cannot be obtained exactly. Therefore, it is essential to conduct a systematical investigation on the variation of electronic and phononic transport properties in GeSe under pressure.

In this study, we investigate the pressure effect on the thermoelectric properties of GeSe based on the first-principles calculations. The lattice thermal conductivity is obtained through the iterative solution of the Boltzmann transport equation, while the electronic transport properties are calculated based on the semi-classical Boltzmann theory. It is observed that the lattice thermal conductivities along the three principal crystallographic directions vary anomalously with pressure. Owing to the pressure-induced enhancement of electrical conductivity, the overall *ZT* values of GeSe are improved significantly. The underlying mechanism is explored in detail by the phonon spectrum and electronic band structures analysis.

## Results and Discussion

**Figure 1:**
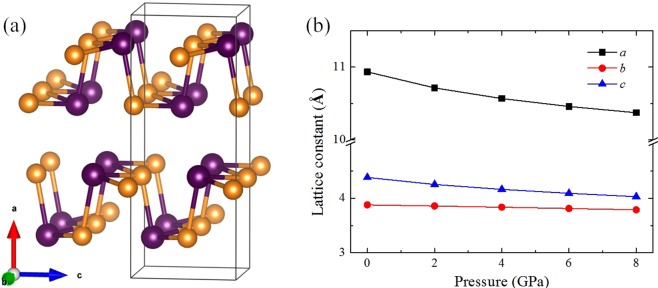
(**a**) Crystal structure of the orthorhombic GeSe (space group *Pnma*, No. 62), where the black border indicates the primitive cell. Ge and Se atoms are shown in purple and orange, respectively. (**b**) Calculated lattice constants of GeSe at different pressures.

At low temperature and ambient pressure, the GeSe crystal adopts a hinge-like layered orthorhombic structure with a *Pnma* space group, as shown in Fig. 1(a). Within the plane of the two-atom-thick slab, each atom is covalently bonded to three nearest neighbors forming zigzag chains along the *b* axis and armchair chains along the *c* axis. These two-atom-thick slabs stack together along the *a* axis by weak van der Waals interactions. Figure 1(b) presents the variations of the lattice constants with pressure. As can be seen from Fig. 1(b), the fully relaxed lattice constants at 0 K and 0 GPa are *a* = 10.934 Å, *b* = 3.878 Å, and *c* = 4.382 Å, which show well agreement with experimental results of low-temperature *Pnma* GeSe crystal^5,26,27^. The lattice constants along all three directions decrease with increasing pressure, while the length reduction along the *a* direction is more significant than that along the *b* and *c* directions. The change of lattice constants with compression is consistent with previous x-ray-diffraction measurements^27^, which indicates that the length along the *a*-axis direction is more compressible than along the *b* and *c* directions. It is known that increasing temperature causes a first-order phase transition from orthorhombic structure to NaCl-type structure at ~900 K^5,28^. In addition, the high pressure stability of orthorhombic phase GeSe up to 9 GPa is verified by lattice dynamics computations^29^. Considering the phase-transition temperature and pressure above, in this work, we focus on the thermoelectric properties of low-temperature orthorhombic phase GeSe below 8 GPa.

**Figure 2:**
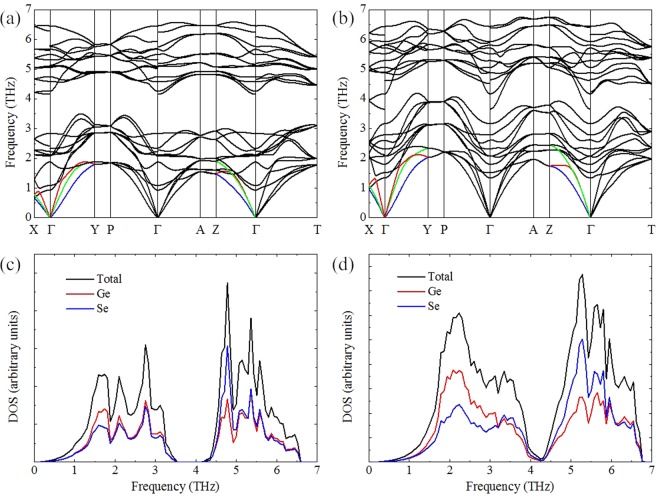
Calculated phonon dispersion curves of GeSe at (**a**) 0 GPa and (**b**) 8 GPa. The TA, TA′, and LA phonon modes are highlighted by blue, green, and red lines along the crystallographic *a*, *b*, and *c* axes, respectively. The total and partial phonon densities of states of GeSe at (**c**) 0 GPa and (**d**) 8 GPa.

The phonon dispersion curves obtained from the harmonic force constants are plotted in Fig. 2(a,b) for two representative pressures. The two transverse acoustic phonon branches (TA and TA′) and longitudinal acoustic phonon branch (LA) are highlighted by blue, green, and red lines along the three principal crystallographic directions. To accurately predict the phonon frequencies for polar materials, the effects of dipole-dipole interactions are incorporated into the dynamic matrix based on the Born effective charges and high-frequency dielectric constants, which can result in the LO-TO splitting (splitting between longitudinal and transversal optical phonon frequencies) presented at the *Г* point. From a comparison of phonon dispersion curves at different pressures, we can clearly see that most phonon modes shift to higher frequencies indicating larger group velocity. Another obvious feature is that a pronounced frequency gap exists in the phonon dispersion at 0 GPa, which separates the phonon spectrum into two discernible regions, each with twelve phonon branches. Under pressure of 8 GPa, the frequency gap disappears due to the hardened low-lying optical phonon modes. This evolution of phonon dispersion with pressure agrees well with previous theoretical computation^29^. From the phonon densities of states shown in Fig. 2(c,d), this change can be seen more clearly. The pressure-induced close-up of frequency gap between low-lying and high-lying optical phonons and the mixing of acoustic phonons with low-lying optical phonons might bring significant modulation to the lattice thermal conductivity, rising from the strong coupling between acoustic and low-lying optical phonons and the increasing probability of optical phonons participating in three-phonon scattering process.

**Figure 3:**
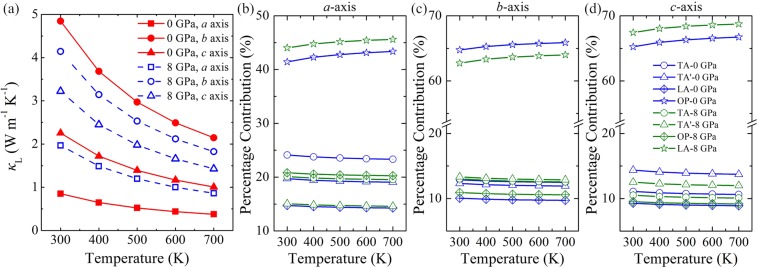
(**a**) The lattice thermal conductivity of GeSe along the *a*, *b*, and *c* directions at two representative pressures from 300 to 700 K. The percentage contributions to the lattice thermal conductivity of GeSe from three acoustic phonon branches and total optical phonon branches along the (**b**) *a* direction, (**c**) *b* direction, and (**d**) *c* direction under different pressures.

Figure 3(a) shows the temperature-dependent lattice thermal conductivity of GeSe along the *a*, *b*, and *c* axes under two typical pressures. From Fig. 3(a), it can be clearly seen that the intrinsic lattice thermal conductivity of GeSe is very low and and highly anisotropic. The lattice thermal conductivity decreases with the increase of temperature following the well-known *κ*_L_ ~ 1/*T* relationship. At 0 GPa, the calculated room-temperature lattice thermal conductivity of GeSe along the *a*, *b*, and *c* directions are 0.85, 4.85, and 2.26 W/mK, respectively, and *κ*_L_ reduces to 0.38, 2.15, and 1.01 W/mK along the corresponding directions as the temperature rises to 700 K. The magnitude sequence *κ*_L,*b*_ > *κ*_L,*c*_ > *κ*_L,*a*_ is consistent with the finding in *Pnma* phase SnSe^4,15,30^, which is attributed to the different phonon group velocities along the three directions, as evidenced by the phonon dispersion. The calculated room-temperature lattice thermal conductivity at 0 GPa is higher than the theoretical predicted value of ~0.64 W/mK along the *b* direction at 300 K^6^. This is understandable that the lattice thermal conductivity obtained by Hao *et al*.^6^ is based on the Debye-Callaway model, this simple model has low computational cost but does not take into account the contributions from optical modes. As discussed later, the optical contributions are as high as those of acoustic modes and cannot be overlooked, thus, the lattice thermal conductivity is significantly underestimated by the Debye-Callaway model^25^. However, our results of the average lattice thermal conductivity is comparable to the experimental values (~1.8 W/mK)^8^ for polycrystalline GeSe samples at room temperature. At a pressure of 8 GPa, the calculated lattice thermal conductivity values along the *a* and *c* directions are larger than those at 0 GPa over the considered temperature range, whereas a surprising reduction in the lattice thermal conductivity along the *b* direction is observed. Specifically, the lattice thermal conductivities along the *a* and *c* directions increase by a factor of 2.31 and 1.43, respectively, while the lattice thermal conductivity along the *b* direction reduces by 15%. As far as we know, Meng *et al*.^31^ also reported an increase in the cross-plane lattice thermal conductivity along with a decrease in the in-plane lattice thermal conductivity in multilayer MoS_2_ under hydrostatic pressure by first-principles calculations. This anisotropic evolution of lattice thermal conductivity with pressure is the result of the competition between the enhanced group velocity and reduced phonon lifetime, which will be discussed in more detail below. Moreover, an important factor that must be noted is the limitations of the method adopted to calculate lattice thermal conductivity. The inputs of second-order and third-order IFCs are determined from zero-temperature ground-state systems, which actually are temperature dependent. Romero *et al*.^32^ have pointed out the necessity of the complete anharmonic behavior at finite-temperature structures when dealing with strongly anharmonic systems. Currently, it is still very challenging to treat strong anharmonic effects when higher order scattering can not be ignored^32,33^. As a result, our calculations only give the qualitative trends of temperature- and pressure-dependent lattice thermal conductivity.

In Fig. 3(b–d), we further show the percentage contributions from different phonon branches to the total lattice thermal conductivity along the *a*, *b*, and *c* directions at different pressures. It can be seen that optical phonon modes make a non-negligible contribution to the total lattice thermal conductivity, especially along the *b* and *c* directions. At a pressure of 0 GPa, optical phonons contribute ~40% to the total lattice thermal conductivity along the *a* direction within studied temperature range, while their contributions to the total in-plane lattice thermal conductivities reach as much as ~65%. The optical phonon contributions for GeSe are comparable to those of SnSe but larger as compared with another group IV-VI compounds PbSe and PbTe with rocksalt crystal structures^30,34,35^, in which the contributions of optical phonons remain about 25% for PbSe and 22% for PbTe between 300 and 700 K. Our calculations indicate that optical phonons are not negligible and play a dominant role in heat conduction of GeSe. Such high optical phonon contributions is the reflection of more optical phonon branches, because GeSe has a large primitive cell with eight basis atoms. On the other hand, the group velocities of optical phonons are large due to more dispersive phonon branches as shown in Fig. 2. At 300 K and 8 GPa, the proportions of optical phonons in the total lattice thermal conductivity along the *a* and *c* directions rise to ~44% and ~68%, respectively, but by way of comparison, the contributions of optical phonons decrease to ~62% along the *b* direction. The results indicate that it is the variation of optical phonon modes that governs the abnormal pressure-dependent lattice thermal conductivity along the three directions.

Next, to reveal the underlying mechanisms governing the phonon transport of GeSe, we analyze the pressure dependence of mode-level phonon properties as a function of frequency at room temperature in terms of group velocity, phonon lifetime, three-phonon phase space, and mode Grüneisen parameter. First, the changes in group velocity along the three directions are plotted (see Supplementary Fig. S1). It is can be seen that, along the *a* direction, the group velocities of acoustic phonons are obviously larger than those of most optical phonons, as evidenced by phonon dispersion characterized by flatted optical branches along the *Γ*-*X* direction. With regard to the pressure effect, pressure enhances the group velocities in all three directions, which facilitates phonon transport in GeSe. However, the variation of group velocity along the *b* direction is minor as compared to those along the *a* and *c* directions.

**Figure 4:**
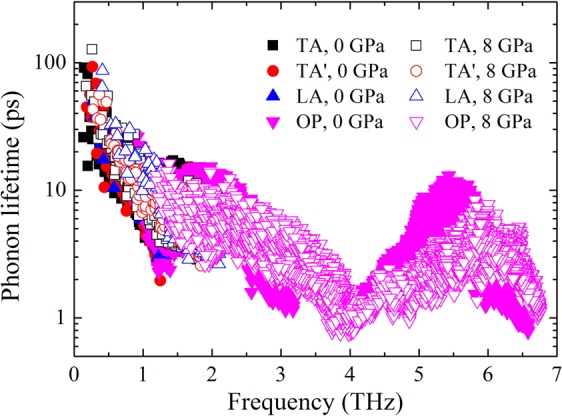
Comparison of the frequency dependent phonon lifetime. The TA, TA′, LA, and OP denote two transverse acoustic, longitudinal acoustic, and optical phonon modes, respectively.

The frequency dependent phonon lifetimes at two typical pressures are displayed in Fig. 4. It is found that most phonon lifetimes are on the order of several picoseconds, which is short enough for low phonon thermal conductivity. Moreover, many optical phonon lifetimes are comparable with those of acoustic phonons. In corporation with large group velocities and broad phonon densities of states, the contribution of optical phonons to the total lattice thermal conductivity overwhelms that of each acoustic phonon branches. Through the comparison of phonon lifetime between 0 GPa and 8 GPa, it can be found that there is a slight decrease in phonon lifetime with pressure, especially for high frequency optical phonons. The phase space for the three-phonon scattering processes, which provides a measure of the available channels for three-phonon processes, is compared between 0 GPa and 8 GPa (see Supplementary Fig. S2). Larger phase space for three-phonon processes implies a larger number of available scattering channels. Consequently, there is an inverse relationship between phase space and the intrinsic lattice thermal conductivity. Three-phonon scattering processes can be further dissociated into absorption and emission three-phonon processes (see Supplementary Fig. S3). For low frequency phonons, absorption channels like A + A → A and A + O → O are dominant processes, while emission channels like O → A + A and O → A + O are anticipated to play an important role at high frequency, where “A” and “O” refer to acoustic and optical phonon modes, respectively. As we can see, the changing trend of three-phonon phase space with pressure is consistent with the variation of phonon lifetime, suggesting the governing role affecting the phonon lifetime. In the inset of Supplementary Fig. S2, the mode-dependent phase space normalized to the zero pressure values is depicted. In the region of 1.0~3.0 THz and 5.0~6.0 THz, the scattering channels are significantly enhanced at high pressure, while it is restricted for the rest frequency region. The main reason for the increased scattering channels is ascribed to more dispersive optical phonons and the close-up of frequency gap between low-lying and high-lying optical phonons. At the frequency ranging from 1.0 THz to 3.0 THz, acoustic phonon and low-frequency optical phonon can easily emerge into one high-frequency optical phonon due to the dispersion of low-lying optical phonon modes considering the energy and momentum conservation criterion. While at the frequency ranging from 5.0 THz to 6.0 THz, there are more chances for a high-frequency optical phonon scattering into two low-frequency phonons. As a result, the phonon lifetime shows a remarkable decrease at high pressure in the corresponding frequency range where the three-phonon scattering channels are enhanced.

The mode Grüneisen parameters of GeSe are calculated (see Supplementary Fig. S4), which can be used to measure the strength of anharmonic phonon-phonon scattering. We can see that the effect of pressure on the mode Grüneisen parameters is not significant with the exception of slight increase for high frequency optical phonons. The mode weighted accumulative Grüneisen parameter is calculated based on $\bar{\gamma }={\sum }_{\lambda }{c}_{{\rm{ph}},\lambda }{\gamma }_{\lambda }/{\sum }_{\lambda }{c}_{{\rm{ph}},\lambda }$, where *c*_ph_ is phonon volumetric specific heat, *γ* is mode Grüneisen parameter, and *λ* denotes a phonon mode with a branch index *s* and a wave vector *q*. The calculated mode averaged Grüneisen parameters are 1.59, 0.92, and 1.16 along the *a*, *b*, and *c* directions at 300 K and 0 GPa, respectively. Previously, Hao *et al*.^6^ has reported higher Grüneisen parameters for GeSe ${\bar{\gamma }}_{a}=4.4$, ${\bar{\gamma }}_{b}=1.9$, and ${\bar{\gamma }}_{c}=2.6$, which are averaged over only the acoustic phonons. Our results of Grüneisen parameters are comparable with those of SnSe (${\bar{\gamma }}_{a}=2.12$, ${\bar{\gamma }}_{b}=1.55$, ${\bar{\gamma }}_{c}=1.66$) and SnS (${\bar{\gamma }}_{a}=2.17$, ${\bar{\gamma }}_{b}=1.44$, ${\bar{\gamma }}_{c}=1.55$) obtained by the same method^34^. At 8 GPa, the mode averaged Grüneisen parameters of GeSe are ${\bar{\gamma }}_{a}=1.78$, ${\bar{\gamma }}_{b}=1.12$, and ${\bar{\gamma }}_{c}=1.38$, respectively, which are slightly larger compared with the zero pressure values. As suggested by the semi-empirical theory, the phonon lifetime due to phonon-phonon Umklapp scattering is inversely proportional to the square of Grüneisen parameter^36^. Therefore, the anharmonic phonon scattering is enhanced by large Grüneisen parameter, which partially contributes to the reduced phonon lifetime at high pressure.

**Figure 5:**
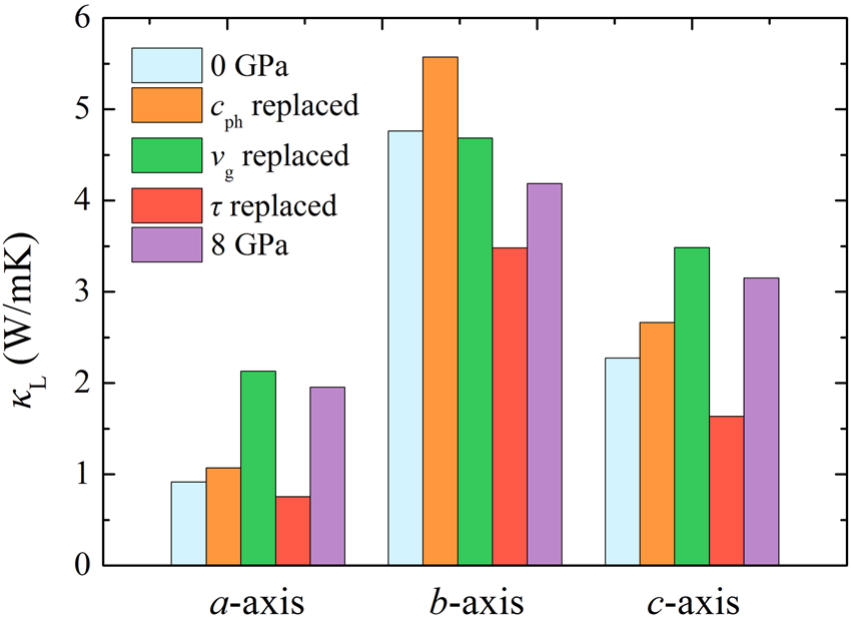
Cross-calculated lattice thermal conductivity with specific heat, group velocity, and phonon lifetime replaced by high pressure values.

To evaluate the relative importance of the phonon specific heat (*c*_ph_), group velocity (*υ*_g_), and phonon lifetime (*τ*) to the lattice thermal conductivity, ${\kappa }_{{\rm{L}},\alpha }={\sum }_{\lambda }{c}_{{\rm{ph}},\lambda }{\upsilon }_{\alpha ,\lambda }^{2}{\tau }_{\lambda }$, we substitute these values of uncompressed state with their respective values at a pressure of 8 GPa. For example, for the case of *c*_ph_ replaced thermal conductivity, the phonon specific heat at 8 GPa is adopted to calculate the total thermal conductivity while fixing group velocity and phonon lifetime at 0 GPa. The replaced lattice thermal conductivity results of GeSe along different directions are shown in Fig. 5, together with unsubstituted values at 0 GPa and 8 GPa. It can be seen clearly that the reduction of phonon lifetime leads to a lower lattice thermal conductivity along all directions, while the enhanced phonon specific heat brings out the augmentation of the lattice thermal conductivity. By comparison, the positive contribution of group velocity to the *a*-axis and *c*-axis lattice thermal conductivity is more significant than that of phonon specific heat. However, the group velocity replaced *b*-axis lattice thermal conductivity shows a very little decrease under high pressure. This is consistent with our previous discussions about group velocity and phonon lifetime. Therefore, the increased group velocity and phonon specific heat along the *a* and *c* directions surpass the reduction of phonon lifetime, which finally results in enhanced lattice thermal conductivity along these two directions under pressure, whereas the insensitive pressure dependent group velocity and depressed phonon lifetime cancel out the increased phonon specific heat, leading to a lower lattice thermal conductivity along the *b* direction at 8 GPa.

Now, we turn our attention to the effect of pressure on the electronic transport properties in GeSe. Applying the semiclassical Boltzmann transport theory, we calculate the thermoelectric transport properties of GeSe under different pressures. The experimentally measured Hall transport properties of Ag-doped GeSe below 700 K suggest that the carrier concentration is ~1 × 10^18^ cm^−3^ ^8^. Therefore, in our transport calculations we consider a hole concentration of 1 × 10^18^ cm^−3^.

**Figure 6:**
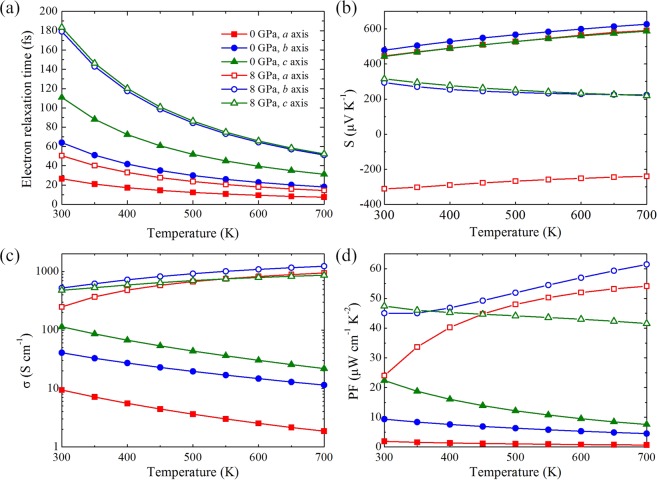
(**a**) Electron relaxation time, (**b**) Seebeck coefficients, (**c**) electrical conductivities, and (**d**) power factors along the *a*, *b*, and *c* axes as a function of temperature at 0 GPa and 8 GPa with a hole concentration of 1 × 10^18^ cm^−3^.

In the output of BoltzTraP code, the electrical conductivity is scaled by the constant relaxation time of unity (1 fs). To overcome this problem and have a comparative assessment of the effect of pressure, we calculate the electron relaxation time approximated by the the single parabolic band (SPB) model at given temperature and carrier concentration. Differently from MoS_2_ and Mg_2_Sn in which the valence band maximum (VBM) is at the *Γ* point^17,19^, in our model system the VBM is not located at the *Γ* point and there are more than one deformation potentials describing the pressure/strain behavior of the electronic band structure, thus we approximately adopt the deformation potential of the VBM along the *Γ*-*Z* direction for the electron relaxation time calculations. The deformation potential is estimated based on the energy change of the VBM by varying the cell volumes instead of a stretch and a contraction along different axes. The obtained deformation potential at the VBM is comparable with that of SnSe^34^. Another approximation we made is that the same relaxation time is used for the conduction bands as for the valence bands in the calculation. Figure 6(a) shows the evaluated electron relaxation time under pressures of 0 GPa and 8 GPa by the SPB model. Apparently, the electron relaxation time is anisotropic and inversely proportional to temperature. At high temperatures, the electron relaxation time is in the order of several tens of femtoseconds. Hao *et al*.^6^ found that the electron relaxation time along the *b*-axis decreases from 27 to 4 fs as the temperature rises from 300 to 800 K for the hole concentration in the range of 4 × 10^19^–6.5 × 10^19^ cm^−3^. Our results seem reasonable compared to the values in ref.^6^ considering the lower hole concentration we adopted. Under compression, the electron relaxation time increases due to enhanced longitudinal sound velocity and reduced effective mass. It should be noted that the used SPB model only considers acoustic phonon scattering, and previous works had demonstrated the dominant role of longitudinal optical (LO) phonon scattering, such as in *n*-type PbTe^37–39^. We roughly evaluate the effect of LO phonon scattering based on the first-principles results of electron-phonon scattering in SnSe^40^, whose electron-phonon scattering mechanisms may be analogous to GeSe due to the structural and chemical similarities (see Supplementary Information for details). Assuming 60% of the total scattering rate is contributed by the LO phonons in GeSe, the electron relaxation times will reduce to 11 fs, 26 fs, and 44 fs along the *a*, *b*, and *c* directions at 300 K and 0 GPa. The electron relaxation times accounting for LO phonon scattering are still in the same order as the results of the SPB model. We also plot the electrical conductivity and power factor scaled by the electron relaxation time as shown in Fig. S5. The changing trends of *σ*/*τ*_*e*_ and *S*^2^*σ*/*τ*_*e*_ are in accordance with the results based on the SPB model. Therefore, although the SPB model relying only on acoustic phonon scattering is certainly not rigorous, it is easy to apply and can to some extent reveal the effect of temperature and pressure on the electron relaxation time and thermoelectric transport properties.

Figure 6(b) plots the Seebeck coefficient as a function of temperature at different pressures. At 0 GPa, the Seebeck coefficients of the three directions increase with increasing the temperature and the values are all above +450 μV/K, suggesting the *p*-type property. Under a pressure of 8 GPa, the absolute values of Seebeck coefficients of the three directions are slightly lower than those at zero pressure and decrease with the increase of temperature. Interestingly, the in-plane direction remains positive Seebeck coefficients (*p*-type), while the Seebeck coefficient along the *a* direction exhibits a sign change, implying a carrier change from *p*-type to *n*-type under pressure. In Fig. 6(c), we show the temperature dependent electrical conductivity under pressure. Opposite to negative pressure trend in Seebeck coefficient, the electrical conductivity is significantly enhanced under pressure. The decreasing Seebeck coefficient and increasing electrical conductivity with temperature at 8 GPa are due to the bipolar transport and originate from the thermal excitation of carriers, which is the result of smaller band gap under pressure as discussed below. Combining the results of Seebeck coefficient and electrical conductivity, the corresponding power factor can be assessed. As shown in Fig. 6(d), because of the boost in electrical conductivity, the power factor has a pronounced increase under pressure. For example, at 0 GPa and 700 K, the calculated power factors along the *a*, *b*, and *c* directions are observed to be about 0.65, 4.48, and 7.55 μW cm^−1^ K^−2^, respectively, and increase to 54.15, 61.45, and 41.57 μW cm^−1^ K^−2^ at 8 GPa and 700 K along the corresponding directions.

**Figure 7:**
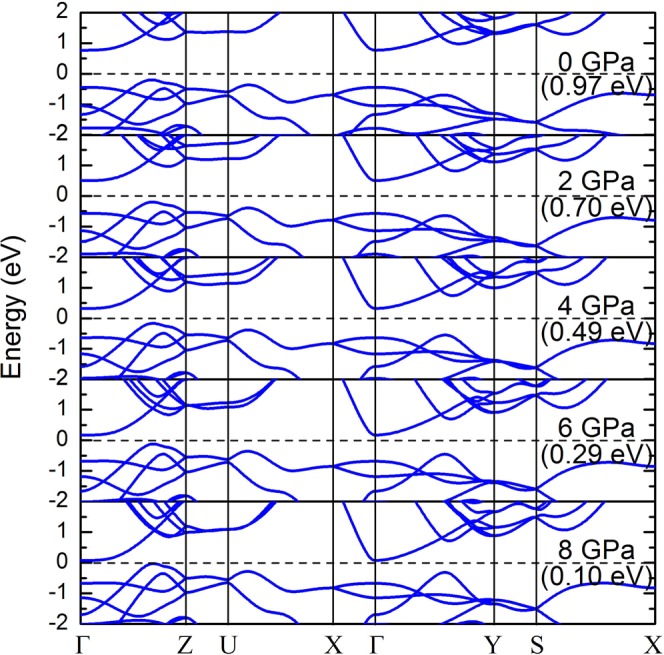
The band structures of GeSe under different pressures.

The large enhancement in thermoelectric properties of GeSe can be understood in terms of the band structure of this material. In Fig. 7, we present the band structures of GeSe at different pressures. The Fermi level is set at 0 eV and is indicated by the black dashed lines. The effect of spin-orbit coupling (SOC) is ignored. Although many works^41,42^ have demonstrated the significant influence of SOC on thermoelectric properties by causing band splitting of the electronic structures, no spin-orbit splitting is expected in bulk group-IV monochalcogenides, because the inversion symmetry together with time-reversal symmetry require that the bands for the two spins are degenerate^43^. Therefore, the SOC barely changes the band shape of GeSe, and electronic transport properties should remain unchanged. From the band structure at 0 GPa, the valence band maximum lies in the *Γ*-*Z* direction while the conduction band minimum (CBM) locates at the *Γ* point, yielding a indirect band gap of 0.97 eV, which is slightly lower than the experimental value of 1.1 eV^44,45^. With the increase of pressure from 0 to 8 GPa, there is a reduction in band gap along with the downward trend of conduction band at the *Γ* point. At higher pressures we notice that the valence band along the *Γ*-*Y* direction moves toward the Fermi level and the flattening of this band is reducing, which is an indication of a light band. The high dispersion in the valence band along the *Γ*-*Y* direction results in the low density of states around the Fermi level but high carrier mobility, thus leading to the decrease of the Seebeck coefficient along with the increase of the electrical conductivity. Along the *Γ*-*X* direction, the valence bands are all below −0.5 eV while the conduction bands become highly dispersive at high pressures. Since the reduction of band gap under pressure, this makes thermal excitation easier and the conduction bands along the *Γ*-*X* direction are occupied with thermally excited electrons, thus displaying *n*-type property along the *a* direction in the real space.

**Figure 8:**
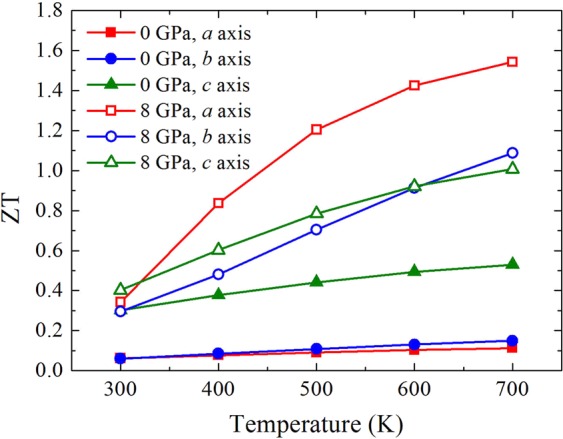
The figure of merit ZT of GeSe along the *a*, *b*, and *c* axes as a function of temperature at 0 GPa and 8 GPa.

Using the above calculated Seebeck coefficient, electrical conductivity, and lattice thermal conductivity, we could estimate the figure of merit by $ZT=\frac{{S}^{2}}{{L}_{{\rm{o}}}+({\kappa }_{{\rm{L}}}/\sigma T)}$, where *L*_o_ = 2.45 × 10^−8^ W Ω K^−2^ is the Lorentz number. The obtained *ZT* values along the three directions at different pressures are shown in Fig. 8 as a function of temperature. The *ZT* values increase as the temperature rises from 300 to 700 K and increasing the pressure causes the enhancement in *ZT* values in all directions, which are attributed to the temperature-dependent and pressure-dependent electrical conductivity. At 700 K and ambient pressure, the computed *ZT* values along the *a*, *b*, and *c* directions are 0.11, 0.15, and 0.53, respectively, and these results are reasonable as compared to previous experimental study, which reported a *ZT* value of ~0.2 for polycrystalline GeSe at the same temperature and carrier concentration^8^. At 700 K and 8 GPa, the estimated *ZT* values along the *a*, *b*, and *c* directions reach 1.54, 1.09, and 1.01, respectively. By the application of hydrostatic pressure, the *p*-type *ZT* along the *b* and *c* directions increase by 7.3 and 1.9 times, while the pressure-induced *n*-type *ZT* along the *a* direction increases by a factor of 14, which is much more prominent than the rise in the in-plane direction. For comparison, Guo *et al*.^46^ reported the thermoelectric properties of monolayer GeSe with a maximum *n*-type *ZT* of 0.8 at 900 K based on the calculated lattice thermal conductivities from Qin *et al*.^47^. In addition, a much higher *ZT* of 1.99 is predicted for monolayer GeSe^48^. It can be seen that the *ZT* values of bulk GeSe at high pressure are comparable to those of monolayer counterpart.

## Conclusions

In summary, we have performed detailed first-principles calculations to investigate the effect of hydrostatic pressure on the electronic and phononic transport properties of GeSe. The lattice dynamics results show that the pressure has profound influence on the phonon dispersion, which exhibits decreasing frequency gap and highly dispersive phonon branches at higher pressures. The results from iterative solutions of phonon Boltzmann transport equation suggest that the lattice thermal conductivities along the three principal crystallographic directions vary differently. The lattice thermal conductivities along the *a* and *c* directions increase with pressure, while an opposite trend is observed in the *b* direction. A detailed analysis on the phonon related properties reveals that there is an evident increase in both phonon specific heat and group velocity under compression with the exception of pressure insensitive group velocity along the *b* direction. The phonon lifetime is significantly reduced at higher pressures due to the enhancement of three-phonon scattering channels and lattice anharmonicity. Along the *a* and *c* directions, the magnitude of pressure-enhanced phonon specific heat and group velocity overwhelm the reduced phonon lifetime, thus resulting in higher lattice thermal conductivities under pressure. For the lattice thermal conductivity along the *b* direction, the largely reduced phonon lifetime in combination with the pressure insensitive group velocity lead to a slight decrease at 8 GPa. From the discussion of the contribution from each phonon branch, it is found that optical phonon branches have considerable contributions to the total lattice thermal conductivity and their pressure variations dominate the diverse pressure-dependent lattice thermal conductivity along the three directions. The results of electronic transport properties demonstrate that the power factors are greatly enhanced due to the boost of electrical conductivity. At 8 GPa and 700 K, the estimated *ZT* values for *p*-type doping along the *b* and *c* directions are about 7.3 and 1.9 times higher than those at ambient pressure. The predicted highest *ZT* value for *n*-type doping along the *a* direction is greater than 1.5 at experimental carrier concentration, which is enhanced by a factor of 14 as compared with the *ZT* value at 0 GPa. Our calculations show that there is plenty of room for improvement of the thermoelectric performance in layered orthorhombic GeSe through the modulation of hydrostatic pressure.

### Computational methods

All the first-principles calculations are carried out based on density functional theory (DFT) as implemented in the Vienna ab-initio Simulation Package (VASP)^49^. The projector-augmented wave (PAW) pseudopotentials^50^ are used to describe the interaction among atoms and the generalized gradient approximation (GGA) in the Perdew-Burke-Ernzerhof (PBE)^51^ form is chosen as the exchange-correlation functional. The kinetic energy cutoff of the plane-wave function is set to 500 eV for all the calculations. The convergent criterion for the total energy difference between two successive self-consistency steps is 10^−8^ eV and the primitive cell is fully relaxed until the maximum force acting on each atom is less than 10^−8^ eV Å^−1^. A well-converged Monkhorst-Pack^52^ *k*-point grid of 5 × 15 × 13 is used to sample the first irreducible Brillouin zone. In general, GGA usually overestimates the lattice constants of weakly bonded layered solids. To include the van der Waals interaction in layered GeSe, the non-local van der Waals density functional termed as optB86b-vdW^53,54^ is taken into account. Since an accurate estimation of band gap is important for the prediction of thermoelectric transport properties, the Tran-Blaha modified Becke-Johnson (TB-mBJ)^55^ exchange-correlation potential is used to calculate the electronic band structure of bulk GeSe.

The BoltzTraP package^56^ is utilized to calculate the Seebeck coefficient and electrical conductivity of bulk GeSe under different pressures. The BoltzTraP code is based on semi-classical Boltzmann theory in conjunction with rigid band and constant relaxation time approximations, and has been successfully applied to various types of thermoelectric materials^57–60^. The electrical conductivity tensors *σ* and Seebeck coefficient tensors *S* as a function of temperature *T* and chemical potential *μ* are expressed as

1 $${\sigma }_{\alpha \beta }(T,\mu )=\frac{1}{{\rm{\Omega }}}{\int }_{-\infty }^{+\infty }{\sum }_{\alpha \beta }(\varepsilon )[-\frac{\partial f(T,\varepsilon ,\mu )}{\partial \varepsilon }]{\rm{d}}\varepsilon $$

2 $${S}_{\alpha \beta }(T,\mu )=\frac{1}{eT{\rm{\Omega }}{\sigma }_{\alpha \beta }(T,\mu )}{\int }_{-\infty }^{+\infty }{\sum }_{\alpha \beta }(\varepsilon )(\varepsilon -\mu )[-\frac{\partial f(T,\varepsilon ,\mu )}{\partial \varepsilon }]{\rm{d}}\varepsilon $$

where *α* and *β* are Cartesian indices, Ω is the volume of unit cell, *ε* is the band energy, *f* is the Fermi-Dirac distribution function of carriers, ∑_*αβ*_(*ε*) is the transport distribution function and defined as

3 $${\sum }_{\alpha \beta }(\varepsilon )=\frac{{e}^{2}}{N}\sum _{i,k}{\tau }_{i,k}{\upsilon }_{\alpha }(i,k){\upsilon }_{\beta }(i,k)\delta (\varepsilon -{\varepsilon }_{i,k})$$

where the summation is over all bands *i* and over all *k* grid, *τ* is the relaxation time, *υ* is the band velocity. It is noted that the most simple approximation that the relaxation time is independent of both *i* and *k* is adopted for the results. From the above equations, it can be seen that the calculation accounts for both valence band and conduction band. Since $\partial f(\varepsilon )/\partial \varepsilon$ has a finite value only in an energy window about a few *k*_B_*T* around the chemical potential *μ*, the carriers in this energy range contribute most to the transport properties. To get well-converged electronic transport quantities, a finer 15 × 45 × 39 *k*-mesh is selected.

For the phononic transport properties, an iterative self-consistent method is employed for solving the phonon Boltzmann transport equation to calculate the lattice thermal conductivity using the ShengBTE package^61^ with the inputs of second-order (harmonic) and third-order (anharmonic) interatomic force constants (IFCs). Both the harmonic and anharmonic IFCs are computed by the finite difference method. Several independent atomic displacements created by the PHONOPY^62^ program within 2 × 6 × 5 supercells are used for the calculation of harmonic IFCs. In the calculation of the anharmonic IFCs, a 2 × 3 × 3 supercell is constructed in consideration of the computational cost and the cutoff radius is considered up to the 27th nearest neighboring shell for the sufficient involvement of the long-range interactions. Finally, the lattice thermal conductivity is integrated on a well-converged 9 × 27 × 23 phonon *q* grid.

To evaluate the electron relaxation time more accurately at given temperature and carrier concentration, the SPB model is employed. According to the SPB model, the electron relaxation time is calculated by

4 $${\tau }_{e}=\frac{\sqrt{2}\pi {\hslash }^{4}\rho {\upsilon }_{l}^{2}}{3{E}_{{\rm{d}}}^{2}{({m}^{\ast }{k}_{{\rm{B}}}T)}^{3/2}}\frac{{F}_{0}(\eta )}{{F}_{1/2}(\eta )}$$

where *ħ* is the reduced Planck constant, *ρ* is the mass density, *υ*_*l*_ is the longitudinal sound velocity, *k*_B_ is the Boltzmann constant, and *T* is the absolute temperature. *E*_d_ is the deformation potential obtained by ${E}_{{\rm{d}}}=\partial {E}_{{\rm{v}}}/\partial (V/{V}_{0})$, which describes the energy change at the band valley with respect to the variation of volume. A series of volumes varying from 0.985*V*_0_ to 1.015*V*_0_ with a step of 0.005*V*_0_ are used to calculate the band structure, where *V*_0_ is the equilibrium volume. The calculated deformation potentials of GeSe are −11.04 eV at 0 GPa and −13.54 eV at 8 GPa for the valence band maximum, respectively. m* is the effective mass at the band extrema, which is calculated using finite difference method^63^. The obtained effective masses for the valence band maximum are ${m}_{kx}^{\ast }$ = 0.58 *m*_e_, ${m}_{ky}^{\ast }$ = 0.47 *m*_e_, and ${m}_{kz}^{\ast }$ = 0.21 *m*_e_ at 0 GPa, respectively, and are reduced to 0.56 *m*_e_, 0.19 *m*_e_, and 0.15 *m*_e_ at a pressure of 8 GPa along the corresponding directions, where *m*_e_ is the free electron mass. *F*_*x*_(*η*) is the Fermi integral given by

5 $${F}_{x}(\eta )={\int }_{0}^{+\infty }\frac{{E}^{x}}{1+\exp (E-\eta )}{\rm{d}}E$$

where *η* = *E*/*k*_B_*T* is the reduced chemical potential. For the given *T* and *η*, carrier concentration *n* is determined by

6 $$n(T,\eta )=\frac{{(2{m}_{{\rm{d}}}^{\ast }{k}_{{\rm{B}}}T)}^{3/2}}{2{\pi }^{2}{\hslash }^{3}}{F}_{1/2}(\eta )$$

## Supplementary information

Supplementary Information

## Data Availability

The datasets generated during and/or analysed during the current study are available from the corresponding author on reasonable request.
