# Supplementary material for: Tailoring phononic, electronic, and thermoelectric properties of orthorhombic GeSe through hydrostatic pressure
Source: Sci Rep. 2019 Jul 1;9:9490. doi: 10.1038/s41598-019-45949-8 (PMC6603015; doi:10.1038/s41598-019-45949-8)
Supplement: Supplementary file 1 — Supplementary Information [file 41598_2019_45949_MOESM1_ESM.pdf]

Supplementary Information

Tailoring phononic, electronic, and thermoelectric properties of orthorhombic GeSe through hydrostatic pressure

Kunpeng Yuan, Zhehao Sun, Xiaoliang Zhang* and Dawei Tang*

Key Laboratory of Ocean Energy Utilization and Energy Conservation of Ministry of Education, School of Energy and Power Engineering, Dalian University of Technology, Dalian 116024, China.

**Corresponding author. zhangxiaoliang@dlut.edu.cn(X.Z.); dwtang@dlut.edu.cn (D.T.)*

Phonon group velocity

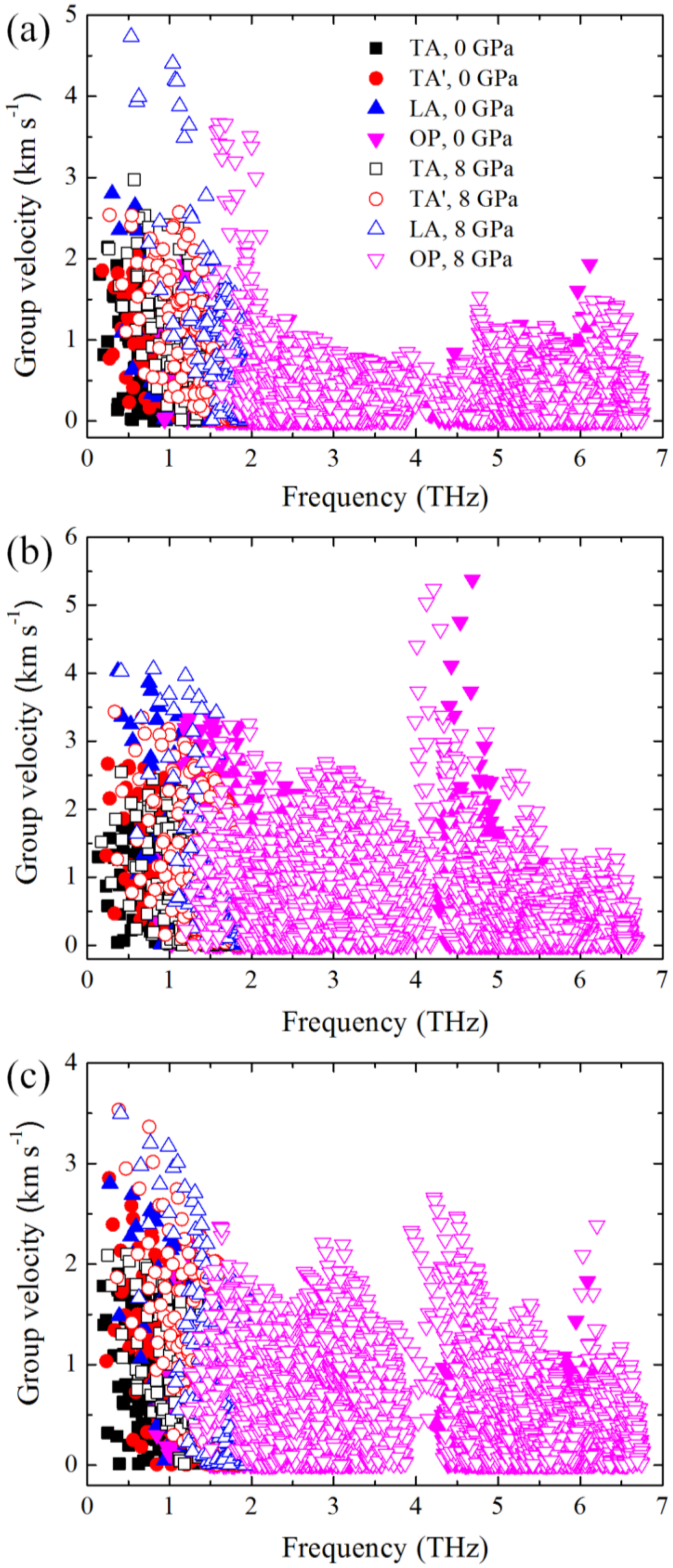

Supplementary Figure S1. Comparison of the phonon group velocity of GeSe along the (a) *a* direction, (b) *b* direction, and (c) *c* direction between 0 GPa and 8 GPa.

Three-phonon scattering phase space

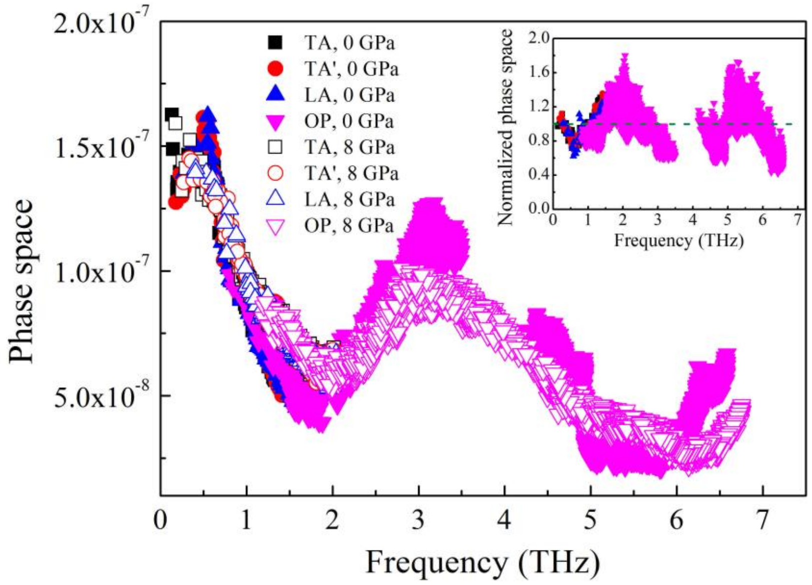

Supplementary Figure S2. Comparison of the frequency dependent three-phonon scattering phase space between 0 GPa and 8 GPa.

To investigate the detailed scattering mechanisms, we plotted the decomposed scattering phase space for absorption and emission processes in Figure S3. It is clear that three-phonon absorption processes predominate in the low-frequency range, while three-phonon emission processes play a dominant role at frequencies above 4 THz. Especially, the absorption processes surpass the emission processes in more than one order of magnitude below 2 THz, and most of the acoustic phonons are in this frequency range together with some low-frequency optical phonons. Therefore, we can deduce that absorption channels like A + A → A and A + O → O are dominant processes for low-frequency phonons. For phonons with frequencies higher than 5 THz, the emission processes are at least one order of magnitude larger than the absorption processes. As a result, the emission channels of high-frequency optical phonons like O → A + A and O → A + O are the main scattering processes. It can also be found that the absorption processes and emission processes are enhanced at 8 GPa compared to those at 0 GPa in the region of 1.0 ~ 3.0 THz and 5.0 ~ 6.0 THz, respectively. Due to the close-up of frequency gap between low-lying and high-lying optical phonons, more optical phonon modes between 3.0 THz and 4.0 THz emerge at 8 GPa, which are absent at 0 GPa. At the frequencies ranging from 1.0 THz to 3.0 THz, one acoustic phonon is more likely to be scattered into a high-frequency optical phonon by interacting with a low-lying optical phonon. Meanwhile, there are more chances for a high-frequency optical phonon ranging from 5.0 THz to 6.0 THz scattering into two low-frequency phonons. These enhanced scattering channels are in consistent with the corresponding frequency ranges where the phonon lifetimes are reduced in Figure 4. For the above reasons, we attributed the reduced phonon lifetime at high pressure to the close-up of frequency gap in phonon dispersion and the coupling between acoustic and low-lying optical modes.

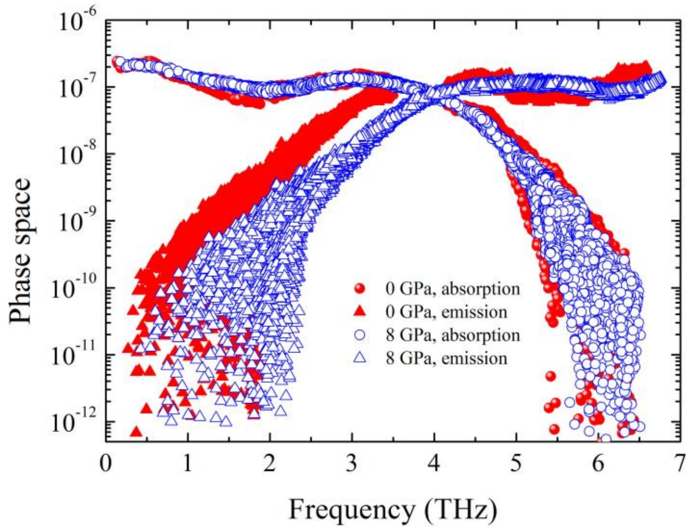

Supplementary Figure S3. Comparison of the frequency dependent three-phonon scattering phase space of absorption and emission processes between 0 GPa and 8 GPa.

Grüneisen parameter

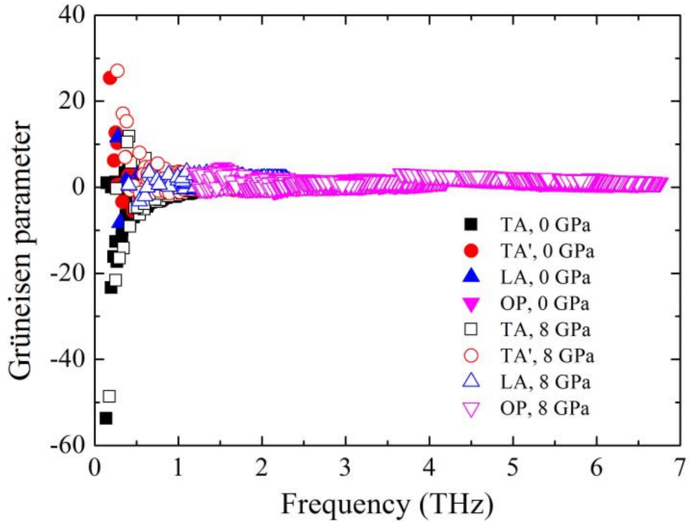

Supplementary Figure S4. Comparison of the frequency dependent Grüneisen parameter between 0 GPa and 8 GPa.

Thermoelectric transport properties

Considering the remarkable contribution from optical phonon scattering, we reconsider the electron relaxation time based on the SPB model. Ma *et al*. investigated the detailed electron-phonon scattering in SnSe, an analog compound with the same crystal structure, and found that the highest longitudinal optical (h-LO) phonons dominate the scattering processes [1]. Their results showed that the total scattering rates are in the range of 10 - 100 THz (corresponding to 10 - 100 fs) at room temperature and the h-LO phonons contribute about 57 - 75 % to the total scattering rate within 0.10 eV away from the band edge. We assume that the electron-phonon scattering mechanisms of SnSe might be analogous to GeSe due to the structural and chemical similarities. Assuming 60% of the

total scattering rate is contributed by the LO phonons in GeSe, the electron relaxation times will reduce to 11 fs, 26 fs, and 44 fs along the *a*, *b*, and *c* directions at 300 K and 0 GPa, which are 40% of the electron relaxation times using the SPB model. The electron relaxation times accounting for LO phonon scattering are still in the same order as the results of the SPB model. To clearly show the effect of pressure on the electronic transport properties, we replot the electrical conductivity and power factor scaled by the electron relaxation time as shown in Figure S5. It can be seen that the changing trends of σ/τ_e and $S^2\sigma/\tau_e$ are in accordance with the results based on the SPB model. Under the assumption of constant relaxation time, the power factors at 8 GPa are 43.4, 4.8, and 3.3 times as large as those at 0 GPa and 700 K, showing significant enhancement in the electronic transport properties by applying hydrostatic pressure.

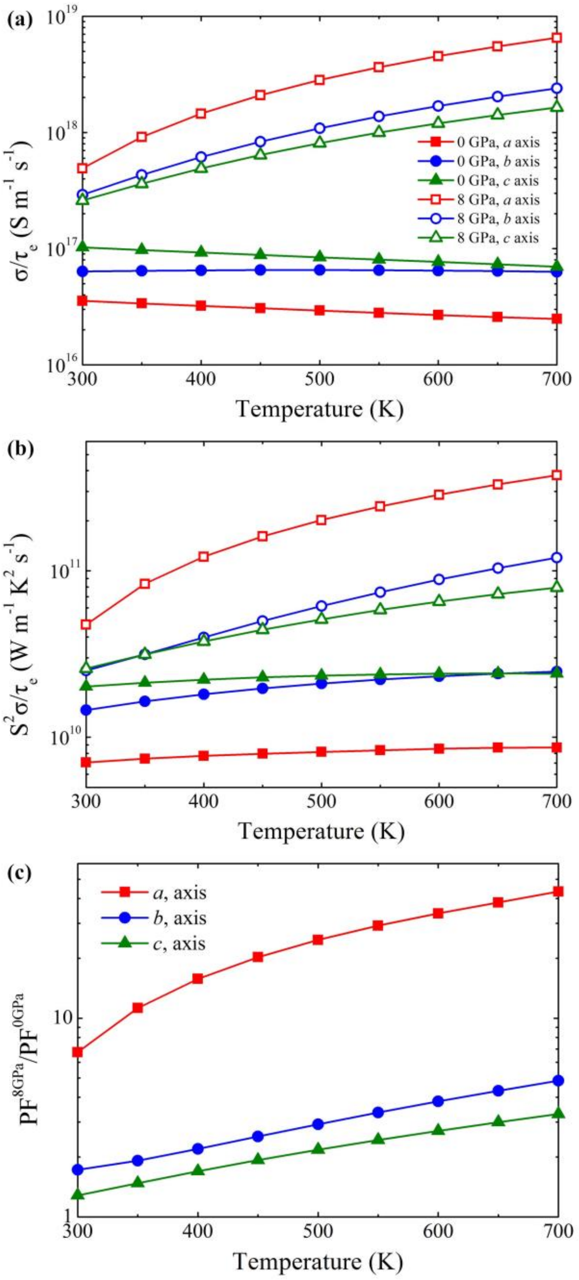

Supplementary Figure S5. Calculated thermoelectric properties of GeSe as a function of temperature at 0 GPa and 8 GPa with a hole concentration of 1×10^{18} cm^{-3}. (a) electrical conductivities, (b) power factors, and (c) the ratio of power factors along the *a*, *b*, and *c* axes.

Supplementary References

1. Ma, J., Chen, Y. & Li, W. Intrinsic phonon-limited charge carrier mobilities in thermoelectric SnSe. *Phys. Rev. B: Condens. Matter Mater. Phys.* **97,** 205207 (2018).
